# Construction of the model for the Genetic Analysis Workshop 14 simulated data: genotype-phenotype relationships, gene interaction, linkage, association, disequilibrium, and ascertainment effects for a complex phenotype

**DOI:** 10.1186/1471-2156-6-S1-S3

**Published:** 2005-12-30

**Authors:** David A Greenberg, Junying Zhang, Dvora Shmulewitz, Lisa J Strug, Regina Zimmerman, Veena Singh, Sudhir Marathe

**Affiliations:** 1 Division of Statistical Genetics, Department of Biostatistics and Psychiatry, Mailman School of Public Health, Columbia-Presbyterian Medical Center, New York, NY 10032, USA

## Abstract

The Genetic Analysis Workshop 14 simulated dataset was designed 1) To test the ability to find genes related to a complex disease (such as alcoholism). Such a disease may be given a variety of definitions by different investigators, have associated endophenotypes that are common in the general population, and is likely to be not one disease but a heterogeneous collection of clinically similar, but genetically distinct, entities. 2) To observe the effect on genetic analysis and gene discovery of a complex set of gene × gene interactions. 3) To allow comparison of microsatellite vs. large-scale single-nucleotide polymorphism (SNP) data. 4) To allow testing of association to identify the disease gene and the effect of moderate marker × marker linkage disequilibrium. 5) To observe the effect of different ascertainment/disease definition schemes on the analysis. Data was distributed in two forms. Data distributed to participants contained about 1,000 SNPs and 400 microsatellite markers. Internet-obtainable data consisted of a finer 10,000 SNP map, which also contained data on controls. While disease characteristics and parameters were constant, four "studies" used varying ascertainment schemes based on differing beliefs about disease characteristics. One of the studies contained multiplex two- and three-generation pedigrees with at least four affected members. The simulated disease was a psychiatric condition with many associated behaviors (endophenotypes), almost all of which were genetic in origin. The underlying disease model contained four major genes and two modifier genes. The four major genes interacted with each other to produce three different phenotypes, which were themselves heterogeneous. The population parameters were calibrated so that the major genes could be discovered by linkage analysis in most datasets. The association evidence was more difficult to calibrate but was designed to find statistically significant association in 50% of datasets. We also simulated some marker × marker linkage disequilibrium around some of the genes and also in areas without disease genes. We tried two different methods to simulate the linkage disequilibrium.

## Background

### The ideas underlying the data simulation

The simulated data for Genetic Analysis Workshop 14 (GAW14) arose out of our experience with analysis of data from several actual diseases and our experience with GAW11. Although the final data contained coarse and fine single-nucleotide polymorphism (SNP) data, microsatellite data, association and linkage disequilibrium elements, the main focus of the simulation was phenotype, a decision we made that was guided by the belief that if the phenotype is correct, reasonable genetic analysis methods will yield answers.

One of the most important emerging questions confronting the genetics of common disease concerns gene × gene interaction and its effect on finding and identifying disease-related loci. This problem constitutes a central theme in the design of the simulation.

### The disease being simulated: Kofendrerd Personality Disorder (KPD)

The clinical characteristics of this non-existent condition were designed to model descriptions, phenotypes, and diagnostic ambiguities that can be found in actual common, and especially psychiatric, disease classification. The extensive description of KPD was designed to reflect the uncertainty, difficulty, and controversy in defining common disease phenotypes. These phenotypes often include sub-clinical, or endo-, phenotypes that are common in the general population but are thought to be related to the disease; however, the connection to disease is often uncertain. We also simulated such endophenotype information, most of which was genetic and related to the disease. Genetic studies of an actual disease often differ in the choice of phenotypic criteria and family structure used for ascertainment. We attempted to model these differences in the four "studies" of KPD that used different criteria to choose the families.

From the point of view of searching for disease genes, KPD and the endophenotypes associated with it probably represent a much stronger genetic/etiologic connection than exists with most actual common diseases, and thus the causative genes and the interactions should be easier to discover.

### Genetic model

**Figure 1:**
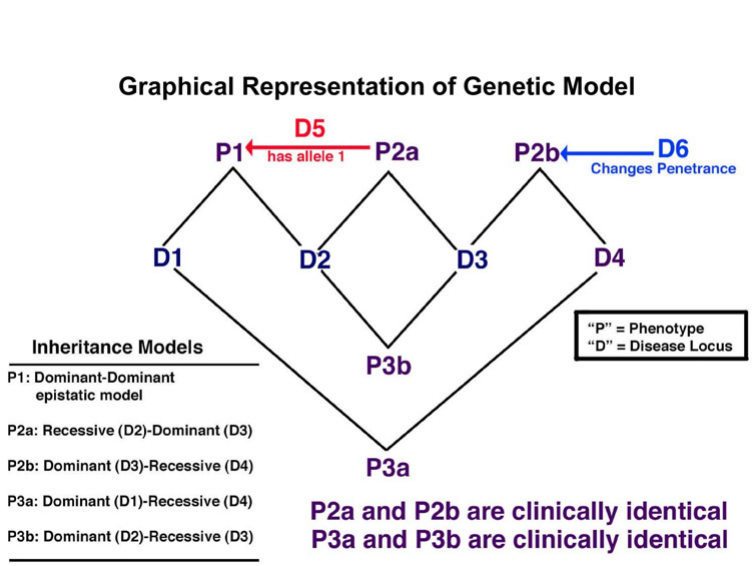
**Graphical representation of the genetic model used in the simulation. **D1-D4 are disease-causing loci. D5 and D6 influence disease expression if the disease genotype is present. P1-P3 are different phenotypes caused by the disease loci to which they are connected by the lines. The "a" and "b" after the phenotype designation indicate identical phenotypes but caused by different genotypes. D5 changes phenotype P2a into P1 when allele 1 is present. D6 changes the penetrance of P2b when allele 1 is present.

The chief elements that went into the genetic model underlying the simulation were:

1. Gene interaction, mostly but not entirely epistatic (see Figure 1).

2. Heterogeneity, both genetic heterogeneity and phenotypic heterogeneity.

3. The presence of "modifier" genes.

4. The presence of subclinical markers or "endophenotypes" that can be seen as a partially penetrant form of the disease. (In this simulation, the endophenotypes can be viewed as a manifestation of a partial disease genotype, e.g., only one of two genes required for trait expression.)

5. The effect of different criteria for diagnosis and different ascertainment schemes based on the beliefs of different investigators.

### Data generation

The program used to generate the family and association data was a modified version of the program used to generate the GAW11 data.

We generated four populations, each with the same underlying population parameters. Three of the populations differed in diagnostic criteria used to ascertain the families. One population was collected solely on the basis of large dense pedigrees.

We extensively tested the linkage-analysis-based detectability of the disease loci using microsatellite data and we aimed at calibrating the parameters to make data analysis appear realistic, based on our experience. This proved a difficult and time-consuming task. However, because the simulation model emphasized gene interaction rather than just detectability, the linkage signals were calibrated to yield evidence of linkage in a majority of data sets at all true loci.

### SNPs, microsatellites, and association

We also included the opportunity for participants to ask questions about microsatellite vs. SNP markers, to pursue "fine" mapping, and to detect association of the disease with SNPs. Because we included control data in the more detailed SNP data (which were not distributed to participants with the coarse SNP and microsatellite data but could be downloaded from the internet), participants had the option of doing family-based or case-control-based association analyses. We did not simulate population stratification.

### Fine SNP data distribution over the internet

We wanted to simulate some actual elements and problems of real data collection, thus our decision to distribute the fine-mapping data, which consisted of 10,000 SNPs over the genome, from a specially-designed web site that limited the number of SNPs that could be requested to an approximation of what one could actually afford in a real research study, using the technology available in 2003. We packaged the chromosomal regions into groups of 20 contiguous SNPs (packets) and allowed participants to download a maximum of 20 packets. Each packet contained files for all replicates from all the populations studied, plus the same chromosomal region from a control population for doing case-control studies. The idea was that participants who did not request answers would first perform linkage analysis on the original data and then focus on those areas that produced the strongest evidence for linkage. Those who did request the answers, we assumed, would also concentrate on those areas in which there were disease-related loci.

### Linkage disequilibrium

One difficulty was how to simulate linkage disequilibrium (LD). We chose to try different approaches to this, including choosing which haplotypes held the disease locus, based on structure and based on frequency. As a model, we used an area of the genome in which there was some marker × marker LD and used the two-SNP haplotype probabilities from that region to simulate the data, modifying the frequencies of the disease allele-carrying haplotypes so that association would be detectable. We tried to calibrate the parameters so that association with SNPs near the disease SNP (which was **not **one of the ones for which there was typing data) would show statistically significant evidence of association in at least 50% of the datasets. In the end, this calibration was difficult and we ran up against the deadline. Thus, we are less sure about the ultimate product for this aspect.

One final note: The original germ of the idea for Kofendrerd Personality Disorder came from a short story by Richard Matheson called "The Creeping Terror" [1]. However, in the original story, the implication was that the condition was environmentally transmitted, not genetic.

### Details of data simulation

#### 1. Overview of the disease model and ascertainment in the data collection groups

As may be noted below, KPD is actually a heterogeneous disease in which four loci are involved. These four loci interact epistatically in a complex way to produce three different but overlapping phenotypes, or "latent traits" (Figure 1, Table 1). There are also two "modifying" loci, one which changes one of the phenotypes into another, and one which affects the penetrance of one of the phenotypes.

There were four "groups" that ascertained KPD data. The Aipotu, Karangar, and Danacaa groups ascertained only nuclear families. The NYC group found mostly three-generation pedigrees with at least four affected members.

A critical aspect of the simulation concerns the ascertainment/diagnostic schemes used to construct the datasets. The Aipotu families were selected when at least two offspring were present who had either Phenotype 1 (P1), Phenotype 2 (P2), or Phenotype 3 (P3) (representing three different phenotypes). Other family members were also counted as affected if they had either P1, P2, or P3. Similarly, the Karangar families were chosen for at least two affected offspring with P2 or P3, and other family members were also counted as affected if they had P2 or P3. The Danacaa families were also collected based on the presence of P1 in two offspring and only P1 was counted as affected in the other family members. These ascertainment/diagnostic schemes were chosen to simulate the variability of ascertainment/diagnosis in actual psychiatric disease. The NYC study chose families based on the presence of at least 4 members affected with P1 or P2 or P3 and at least one of each phenotype was required to be present in each family. This ascertainment fact was deliberately not noted in the data description.

#### 2. Areas with LD/unique characteristics

There is LD between the SNPs of the detailed map (markers named beginning with the letter "B" available from the web site described in the data description) in regions surrounding three of the four major disease loci. There is also LD in four regions that do not contain disease loci.

To generate the LD, we generated 13- or 15-SNP haplotypes based on two-SNP haplotype probabilities derived from actual data from a small region of human chromosome 6. Using these probabilities, we generated between 200 and 2,000 haplotypes. We then inserted the disease gene into a proportion of these haplotypes. We tested whether disease association could be detected with SNPs surrounding the disease mutation and tried to adjust the gene frequencies so that association with the neighboring SNPs could be detected in at least 50% of the datasets. We used two different approaches to choosing those haplotypes destined to be carriers of the disease allele:

1. Haplotypes were sorted, treating the haplotypes as a character string. A group of haplotypes that were similar in structure (that were neighbors after sorting) were chosen to carry the disease allele and the frequency of those haplotypes adjusted to equal the disease gene frequency. This approach was an attempt to model the situation where similar haplotypes carry the disease allele. When the parental genomes were simulated, the haplotypes were chosen randomly, proportional to the frequency of the haplotype in the population, and inserted into the appropriate place in the genome. Recombination and gamete production for the genome followed.

2. After generating 2,000 haplotypes, the haplotypes were sorted by frequency. Then, starting with haplotype number 200, the disease allele was inserted into the haplotypes until the summed frequency of those haplotypes was equal to the population disease gene frequency. This was an attempt to model disease alleles that came into the population less closely tied to haplotype structure compared to the first method. When the parental genomes were simulated, the haplotypes were chosen randomly, proportional to the frequency of the haplotype in the population. Recombination and gamete production for the genome followed.

Region 1 was created without LD, that is, the probabilities of all haplotypes were randomly generated, but we chose a restricted number of haplotypes to act as controls on the procedures we described above. Thirty haplotypes were chosen arbitrarily to carry the disease allele and their frequencies adjusted so that their sum equaled the disease gene frequency.

The locations and method used to generate the haplotypes, and disease-carrying haplotype frequencies are listed below (see also Table 2):

1. Locus D1: SNP loci B01T0554-B01T0567. No LD. There are a total of 500 haplotypes, 30 of which carry a disease allele.

2. Locus D2: SNP loci B03T3056-B03T3068. There is LD in this region. The disease-carrying haplotypes are defined by sorting by haplotype structure and then choosing adjacent haplotypes. 194/1003 haplotypes in this region carry disease alleles. Disease mutation is at the end of the chromosome.

3. Locus D3: B05T4135-B05T4142: There is LD in this region. Disease-carrying haplotypes chosen by similar frequency. 241/1,330 haplotypes carry disease alleles.

4. Locus D4: B09T8331-B09T8342: LD is present. 18/200 haplotypes carry disease alleles.

In addition, the following non-disease-related regions were generated with LD: loci B02T1014-B02T1028, loci B03T2400-B03T2414, loci B08T6239-B08T6253, and loci B08T7272-C08R0670.

#### 3. Phenotypes

There are three underlying latent traits designated P1, P2, and P3 (see Figure 1), the genetic dependencies of which are described in Table 1. In addition, there are 12 subclinical phenotypes or traits that occur in both affected and unaffected family members. Ten of these traits are entirely genetic with reduced penetrance, and two are entirely randomly assigned. The genotype-phenotype relationships of the genetic traits are shown in Table 3.

The following description of the simulated disease was distributed with the original data.

### Description of clinical characteristics of KPD and how the family data were collected and classified

KPD (DSM 301.98.6), first described by Matheson (1959) and sometimes called Pathologischezurueckliegenheit, is a psychiatric syndrome characterized by an apparent overwhelming concern, or even obsession, with the meaning of the patient's inner emotions and world view but at the same time subsuming the emotions of others into the self. Like Narcissism (DSM 301.81), these patients can be easily injured psychologically, but unlike Narcissism, they tend not to be arrogant or haughty. KPD also differs from Narcissism in that the patient is overwhelmingly concerned with the emotional state of his/her acquaintances (sufferers of KPD seldom have what could normally be characterized as friends), but usually only as it relates to the patient's own psychological state. They appear not to distinguish between their own emotions and the emotions of others.

The condition is thought to be genetic in origin, possibly exacerbated by prevailing social conditions.

The frequency of KPD is difficult to estimate, given the ambiguous nature of its symptoms. It has been estimated to occur in as much as 10% of the population, although it appears common in some areas but absent in others. Also, diagnostic criteria vary. The best estimate is that the population prevalence is 3%.

Studies have demonstrated a number of characteristics (phenotypes) which, while apparently disparate, all "tend to" be found to a greater or lesser extent among KPD sufferers and their relatives. The following characteristics have been reported.

1. One hallmark of KPD patients is their constant concern with their emotional state and this concern will be the center of their conversation, as they wish to incorporate or ''connect with'' the emotional states of others. In conversation, topics are always included as they relate to the effect on the emotional state of the patient. This symptom occurs without some of the pathologies seen in, for example, borderline personality disorder (DSM-IV 301.83) or histrionic personality disorder (DSM-IV 301.50). The KPD patient likes to give the appearance of being much less focused on ''self'' and more concerned that every acquaintance share their ''connection'' with each other and be part of the emotional ''community''. It must be emphasized that in actual fact, these communities are imagined, as KPD sufferers appear to ignore what other members of their acquaintanceship actually say.

2. Another manifestation of the "communal" pathology of KPD sufferers is that an unusually high proportion of sufferers may join cults. Those sufferers with greater social skills even "found" such cults. (See the case report by Nash, "The Seven Spiritual Ages of Mrs. Marmaduke Moore")

3. Any humorous stories (jokes) induce strong psychological discomfort and feelings of being threatened, irrespective of quality, subject matter, and humorousness of the jokes themselves. This may arise from the feeling that the telling of jokes removes or sets apart the joke-teller from the imagined communal one-ness, or the discomfort may arise from the perceived social stigma of not understanding the humor (see below under "humor impairment").

4. KPD patients may suffer from a form of humor impairment. They frequently report that they have difficulty understanding what is humorous about some jokes that others find amusing, particularly word-plays. One case report noted that, asked to give an example, a patient said, "What has four wheels and it flies?" instead of, "What has four wheels and flies?" The answer to the former might be, for example, "a Cessna" (a brand of airplane; thus, this is an answer without humor) and the answer to the latter is "a garbage truck", but the KPD sufferer could not understand the difference between the two questions and why one might be amusing and the other not.

5. KPD patients show unusual speech patterns. This includes 1) a tendency to form words in the throat ("toe-tulee" (soft or swallowed L-sound)); 2) frequent use of words that convey no information. It is not unusual for KPD sufferers to make these words the most frequently used in every sentence; 3) an inability for even the apparently well educated to articulate thoughts coherently. An example of an utterance by a typical KPD sufferer: "Like, man, it was totally ya know, dude like, ya know?" Thus, an important element of the differential diagnosis is distinguishing KPD from some forms of mental retardation. When KPD was originally described, these traits were almost pathognomonic for the disease. Unfortunately, these patterns have spread to the general population so that the diagnostic value of the speech pattern trait is much diminished. However, because the speech patterns of the KPD-type in the general population are seen mostly prominently among the immediately post-pubescent, older patients with these patterns may well have KPD.

6. A strong aversion to travel by foot (i.e., walking). Patients will use their vehicles (large, gasoline-consuming behemoths are preferred by KPD suffers) to travel distances in which walking would take less time than driving; thus, speed of travel plays no rational role in the manifestation of this phenotype. Also, a common delusion among patients is that travel to any location always takes twenty minutes. (Note: This tendency was reported to be characteristic as early as the 18th century and is thus one of the earliest-noted symptoms of KPD.)

7. Pathological fear of rain, increasing to horror and terror with the presence of snow. (Paradoxically, this is not true in connection with skiing, in which snow is seen as "tame", "no threat", and which need not be "shoveled").

8. Together with the anxiety caused by weather is an extreme sensitivity to what patients describe as ''cold'' (e.g., 20 degrees Celsius or less) or ''hot'' (27 degrees Celsius or more). But patients can have paradoxical reactions to ambient conditions. Some cannot bring themselves to dress ''appropriately'' for their perceived sensitivity, i.e., they will not wear, and show a strong aversion to, warm clothing. Many prefer to shiver, complain and obsess about the weather, even (or especially) in areas that have no weather. This symptom may be related to thoughts that dwell on body habitus (see below). Others will dress in goosedown clothing in weather the normal population would consider perspiration-inducing. Thus, KPD patients will sometimes arrive for their appointments looking like the ''Michelin Man,'' in their down vests and jackets, even when the weather is balmy, and arrive in swim attire even when the temperature is not particularly high. This latter is seen especially in men with high abdominal muscle definition (see below, under ''body habitus'').

9. A preoccupation with body habitus, both of the patient's own and with that of others. In male sufferers, this can manifest as an extreme concern with definition of abdominal muscles ("washboard abs," "sixpack"). These patients have a notable comorbidity for steroid drug use. In women, this same tendency can manifest as an obsession with body weight, or concern about perceived, often imagined, "defects". Elective surgery or other medical intervention changing body or specific organ appearance/prominence is frequently sought by KPD sufferers, and thus morbidity associated with these operations is more frequent among KPD patients. Female sufferers may exhibit anorexia/bulimia. KPD patients may have a mild form of Body Dysmorphic Disorder (DSM-IV 300.7).

10. Anxiety or panic when innocently approached by a stranger, e.g., for directions. This is the most obvious manifestation of the tendency to avoid interaction with any but the circle of habitual acquaintances.

11. Unusual tolerance, even enjoyment, when surrounded by noxious automobile exhaust. This goes along with

12. An obsession with automobiles (cf, driving obsession, above).

13. A fixation, obsession, or unusual concentration on some popular entertainers. It is generally accepted that this tendency arises because the KPD sufferer has accepted the entertainer, with whom he/she is unacquainted except as seen on television or in the theater/film, as belonging to his/her emotional communal group.

14. Tendency to fiscal irresponsibility on an extremely large scale. (Although this trait is of questionable diagnostic value for an individual patient, it may be useful in recognizing "communities" of sufferers.)

Because of these varied phenotypes, there has been much disagreement about how the disorder should be defined. Nosology for KPD falls into three different classifications; all three, or combinations thereof, have been used to diagnose KPD:

1. The unusual "communal emotionality" that appears in many KPD patients is classified by some investigators as the most important element of KPD. The characteristic pathologies that go into this definition are:

"Communally-shared emotions"

a. Joining/founding cults

b. Fear/discomfort with strangers

c. Dislike of jokes told face to face

d. Obsession with entertainers

e. Humor impairment

Other investigators focus on the behavior-related symptoms:

2. Behavioral-related

a. Fascination with automobiles

b. Aversion to walking

c. Uncommunicative, contentless speech patterns

d. Fiscal irresponsibility

Still others focus on the anxiety-related symptoms:

3. Anxiety-related

a. Morbid anger/fear/terror concerning rain/snow

b. Reluctance to wear clothing appropriate for subjective temperature

c. Body-image concerns/mild body dysmorphic disorder.

As noted above, the syndrome appears common in certain geographical locations and rare in others, or the cluster of symptoms may vary with geography.

### Where and how the data were collected

The data that were available for analysis at GAW14 included data collections from four different groups of investigators. Each of these geographically diverse groups had collected families diagnosed with KPD, but the criteria for diagnosis varied. Patients were classified as KPD based on the diagnosing physician's judgment. In all groups except the NYC group, ascertainment required at least two affected offspring to be present. The NYC group required four affected family members.

1. The group from the country of Aipotu, a populous semi-tropical, semi-desert country, has a high prevalence of KPD. The cases that come to attention in Aipotu run the full gamut of symptoms listed above, from the "communal-emotional", behavioral, and anxiety-related groups. Aipotu also has an unusual number of automobiles per capita and a population who are often considered quite attractive, on the average. These investigators classify only anyone with "notable clusters" of symptoms from any of the groups as KPD. Thus, the families in this dataset were ascertained when at least two siblings could be classified under any of the diagnostic groups or any combination.

2. The country of Karangar, on the other hand, is a highly industrialized, mostly urban island. This island also has a surprising number of religions and only occasional rain or snow, which, when it precipitates, both decreases traffic on the roads and increases traffic accidents, usually caused by sudden slowing of people driving in the fast lane. Elective surgery to improve appearance is frequent. Speech patterns of the KPD-type are thought to be infrequent. On this basis, anxiety symptoms and body-image difficulties figured prominently in diagnoses by Karangar psychiatrists. Thus, families collected by these investigators included only those two types, and individuals with prominent behavioral symptoms were not classified as affected.

3. The community of Danacaa is one of the poorest in the world, despite the extremely high productivity of its inhabitants, its large industrial base, and its high GNP. Due to poor decision-making, the result of electing unqualified but popular neophyte politicians to office, almost its entire GNP goes to servicing the enormous domestic and foreign debt and the country's medical care system. Speech patterns typical of KPD also appear to be relatively common. Investigators at the Danacaa Urban Medical Benefits Institute collected families in which behavioral symptoms were prominent.

4. A fourth group is located in New York City. KPD is virtually unknown in New York, indicating the influence of environment on expression of the disease (people never want to share their feelings in NYC). However, the Genome Research for Elucidation of Effective Drugs Institute in NYC received a generous contract from the KPD Foundation, a non-profit group dedicated to raising awareness of KPD and as much money as they can. The GREEDI researchers chose to collect two- and three-generation pedigrees of KPD with at least four affected members.

In all four data collection efforts, family members not meeting criteria for KDP were tested using the Kofendrerd Research Assessment Protocol and investigators recorded whether unaffected family members had any of the 12 (a-l) categories of clinical characteristics under the three diagnostic groups listed above.

The prevalences of KPD and the traits in the three population-based studies are listed in Table 4.

## Abbreviations

GAW: Genetic Analysis Workshop

KPD: Kofendrerd Personality Disorder

LD: Linkage disequilibrium

P: Phenotype

SNP: Single-nucleotide polymorphism

## Figures and Tables

**Table 1:** Genetic model parameters

	Major loci	Disease allele frequency	Inheritance at loci epistatic models	Modifying genes/penetrance
Phenotype 1	D1 D2	0.015 0.15	Dominant-Dominant	Penetrance of genotype is 0.6
				
Phenotype 2	D2 D3	0.15 0.2	D2-D3 Recessive-Dominant	If D5 has allele 1, Phenotype 2 will be converted into. Phenotype 1 with a 0.7 probability Gene frequency allele 1 at D5 = 0.25.
	D4	0.3	D3-D4 Dominant-Recessive	If D6 has allele 1, penetrance is 0.3, otherwise, penetrance is 0.6. Allele 1 frequency at D6 = 0.08
				
Phenotype 3	D1 D4	0.15 0.3	D1-D4 Dominant-Recessive	Penetrance of genotype = 1.0
	D2 D3	0.15 0.2	D2-D3 Dominant-Recessive	Penetrance of genotype = 0.4

**Table 2:** Location of disease-related loci

Disease locus name	Located between (or at) markers (locus)^a^
D1	C01R0052 and B01T0561
D2	B03T3067 and C04R0282^b^
D3	B05T4136 and C05R0380
D4	C09R0765 and B09T8337
D5	C10R0880^c^
D6	C02R0097^c^

^a^D1-D4 do not appear in the map.

^b^B03T3067 is the last visible SNP on chromosome. The disease locus is the last SNP. So C04R0282 should not be linked to B03T3067 or the disease.

^c^Disease locus is marker.

**Table 3:** Genotype-phenotype relationships for subclinical traits

Trait	Loci involved	Inheritance of trait
Unaffected individuals		
a	D1 + D3	dominant, 30% penetrance
b	D1	dominant, 30% penetrance
c	D4	dominant, 15% penetrance
d	D3	dominant, 18% penetrance
e	D2	dominant, 19% penetrance
f	D2	dominant, 19% penetrance
g	D4	dominant, 15% penetrance
h	D2	dominant, 20% penetrance
i	none	random, 30% probability
j	none	random, 30% probability
k	D2 + D4	one disease allele at each locus, 30% penetrance
l	D4	recessive, 30% penetrance
Affected individuals		
Phenotype 1	D1 + D2	have traits b, e, f, h
Phenotype 2	D2 + D3, D3 + D4	c, d, e, f, g, h
Phenotype 3	D1 + D4, D2 + D3	b, c, d, e, f, g, h
a	D1 + D3	dominant, 100% penetrance
i	none	random, 30% probability
j	none	random, 30% probability
k	D2 + D4	one disease allele at each locus, 30% penetrance
l	D4	recessive, 30% penetrance

**Table 4:** Frequency of KPD and associated traits of populations

	Populations		
	
	Aipotu	Karangar	Danacaa
KPD	0.024	0.019	0.008
a. Joining/founding cults	0.005	0.003	0.005
b. Fear/discomfort with strangers	0.021	0.016	0.015
c. Dislike of jokes told face to face	0.085	0.094	0.078
d. Obsession with entertainers	0.079	0.085	0.068
e. Humor impairment	0.070	0.066	0.058
f. Fascination with automobiles	0.070	0.069	0.055
g. Aversion to walking	0.090	0.093	0.074
h. Uncommunicative, contentless speech patterns	0.074	0.072	0.062
i. Fiscal irresponsibility	0.152	0.156	0.145
j. Morbid anger/fear/terror concerning rain/snow	0.156	0.151	0.152
k. Reluctance to wear clothing appropriate for subjective temperature	0.048	0.045	0.044
l. Body-image concerns/mild body dysmorphic disorder	0.029	0.026	0.027
